# Antifungal susceptibility pattern of *Candida* isolated from cutaneous candidiasis patients in eastern Guangdong region: A retrospective study of the past 10 years

**DOI:** 10.3389/fmicb.2022.981181

**Published:** 2022-08-05

**Authors:** Hazrat Bilal, Bing Hou, Muhammad Shafiq, Xinyu Chen, Muhammad Akbar Shahid, Yuebin Zeng

**Affiliations:** ^1^Department of Dermatology, The Second Affiliated Hospital of Shantou University Medical College, Shantou, China; ^2^Skin and Venereal Diseases Prevention and Control Hospital of Shantou City, Shantou, Guangdong, China; ^3^Department of Cell Biology and Genetics, Shantou University Medical College, Shantou, China; ^4^Department of Pathobiology, Faculty of Veterinary Sciences, Bahauddin Zakariya University, Multan, Pakistan

**Keywords:** cutaneous candidiasis, antifungal resistance, *C. albicans*, retrospective study, China

## Abstract

Cutaneous candidiasis is one of the most prevalent mycotic infections caused by *Candida* species. The severity of infection mounts faster when the species shows antifungal resistance. In the current retrospective study, we aimed to analyze the occurrence, causes of cutaneous candidiasis, and antifungal susceptibility pattern of *Candida* isolates from Skin and Venereal Diseases Prevention and Control Hospital of Shantou, located in eastern Guangdong, China. The laboratory data of all patients (*n* = 3,113) suffering from various skin and venereal infections during January 2012 to December 2021 was analyzed through Excel and GraphPad prism. Our analysis indicate that cutaneous candidiasis was 22.29% (*n* = 694), of which 78.53% (*n* = 554) of patients were males and 21.47% (*n* = 149) of patients were females. The median age of patients with cutaneous candidiasis was 38-year [interquartile range (30–48)]. Most cases occurred in the adult age group (19–50  years). Regarding the species type, the *Candida albicans* were prominently detected (*n* = 664, 95.68%), while non-*C. albicans* were found only in 30 (4.32%) patients, which were *C. glabrata* (*n* = 18), *C. krusei* (*n* = 8), *C. tropicalis* (*n* = 3), and *C. parapsilosis* (*n* = 1). The *C. albicans* susceptibility rate for terbinafine, miconazole, voriconazole, itraconazole, fluconazole, ketoconazole, nystatin, 5-flucytosine and amphotericin B were 10.83, 29.32, 59.39, 78.53, 85.28, 87.75, 99.59, 99.41, and 100%, respectively. Finally, all *C. glabrata* isolates were found susceptible to all tested azole drugs with exception to miconazole against which 8.33% of isolates showed resistance. The findings of this study will help healthcare officials to establish better antifungal stewardship in the region.

## Introduction

Cutaneous candidiasis is a superficial infection of skin and mucus membranes caused by yeast from genus *Candida* (Nurdin et al., 2021). *Candida albicans* is the most common specie responsible for candidiasis in humans; however other species like *C. glabrata*, *C. tropicalis*, *C. krusei*, *C. parapsilosis*, and many others are also causing skin infections (Bhattacharya et al., 2020). The *C. albicans* is an opportunistic yeast that mainly causes infections in immunocompromised patients or those with nutritional deficiencies and endocrine disorders. Besides these, local factors like xerostomia, ulcerations, radiation-induced mucositis, trauma-induced skin damage, and skin maceration increase the morbidity rate (Sadeghi et al., 2019). Types of cutaneous candidiasis are candidal vulvovaginitis, candidal balanitis, congenital candidiasis, candidal diaper dermatitis, oral candidiasis, intertrigo, decubital candidiasis, paronychia, perianal dermatitis, and erosio interdigitalis blastomycetica. Pustules, papules, ulcerations, and vesicles are typical signs of cutaneous candidiasis (Edwards, 2015). A study reported that 7% of all inpatients and 1% of all outpatients’ visits to dermatological hospitals had cutaneous candidiasis (Taudorf et al., 2019). The mortality rate of cutaneous infections is relatively low; nevertheless, if the infections remain enigmatic or untreated for a long time, they might cause systematic and invasive candidiasis, with an approximately 25–50% mortality rate (Tortorano et al., 2021).

The global therapeutic guidelines for rare yeast infections are available, but cutaneous candidiasis remains unaddressed (Chen et al., 2021). Physicians prescribe various topical and oral antifungal agents combined with antibacterial, anti-inflammatory, and corticosteroid drugs for its treatment. Common topical antifungal drugs for cutaneous candidiasis are clotrimazole, nystatin, and miconazole. The terbinafine, ketoconazole, and fluconazole are also studied as systematic therapeutic agents for cutaneous candidiasis (Taudorf et al., 2019). Due to the lack of national guidelines for treating cutaneous candidiasis, the misuse of available antifungal drugs occurs, which endorses antifungal drug resistance (Markogiannakis et al., 2021). Antimicrobial resistance is a worldwide health concern; prolonged hospital stays, increased patient cost burden, and mortality rates (Ur Rahman et al., 2018).

Locally and country-wise antifungal drug resistance surveillance studies need to be performed to depict the current scenario. These surveillance studies will help physicians and healthcare officials to properly manage and treat infections (Badali and Wiederhold, 2019). Therefore, the purpose of the current study was to retrospectively analyze the prevalence of cutaneous candidiasis reported over the past 10 years in the Skin and Venereal Diseases Prevention and Control Hospital of Shantou in eastern Guangdong, China. Furthermore, the current study sorted out candidiasis in different age groups and gender, data about various *Candida* species, and their antifungal drug susceptibility profiles.

## Materials and methods

### Study design

The current retrospective study was conducted at Skin and Venereal Diseases Prevention and Control Hospital of Shantou city, Guangdong, China. Data about cutaneous candidiasis were obtained from laboratory records of the hospital for the past 10 years (January 2012 to December 2021).

### Study variables

The data of all patients with cutaneous infections were obtained for which direct microscopy, candida growth culture, and antifungal susceptibility tests had performed. The patient’s age, gender, date of sample collection, sample type, *Candida* species type, and the antifungal susceptibility profile for each species were obtained from laboratory records and saved in an Excel sheet for further analysis.

### Routine laboratory protocols

In routine, every patient with cutaneous fungal infections was first recommended for direct microscopy with potassium hydroxide to visualize fungal pathogens. The positive samples with the *Candida*-like growth were cultured on CHROMagar*-Candida* medium to examine and identify *Candida* following the standard protocol (Saud et al., 2020). Furthermore, the antifungal susceptibility tests for positive *Candida* cultures were performed using CLSI-recommended broth microdilution methods or ATB fungus-2 kit. From 2012 to 2018, antifungal susceptibility tests were performed for fluconazole, miconazole, terbinafine, ketoconazole, itraconazole, and nystatin according to CLSI broth microdilution method (Espinel-Ingroff, 2022). Onward 2019, the tests were performed by ATB fungus-2 kit, and the tested antifungal agents were 5-flucytosine, voriconazole, fluconazole, amphotericin B, and itraconazole. The susceptibility, intermediated, and resistant results were interpreted according to the CLSI M60 or epidemiological cutoff values guidelines (CLSI, 2017; Procop, 2020).

### Data analysis

The patients’ data were classified into four groups depending on age: infants; less than 1 year of age, pediatrics; aged from 1 to 18 years, adults; aged from 18 to 65 years, and older adults; ages greater than 65. The adult age group was further divided into four groups: group I; 18–30 years of age, group II; 31–40 years of age, group III; 41–50 years of age, group IV; 50–65 years of age. The number and percentage of *Candida* species in each age group, patient gender type, and year of the report were noted. The antifungal susceptibility patterns for each *Candida* species were amalgamated over the past decade. The percentage of susceptible, intermediate, and resistant *Candida* species against the examined antifungal drugs was determined.

Moreover, the year-wise antifungal susceptibility pattern of *C. albicans* was determined. The trend of year-wise susceptibility patterns of fluconazole and itraconazole were resolved. The data numeration and percentages were calculated by Microsoft Excel 2016, while the statistical analysis and graphs constructions were performed by GraphPad prism v.8.0 software. The total number of *C. albicans* cases occurred each year, and the number of cases in different age groups of patients were compared using student’s *t*-tests. Furthermore, gender base significance was calculated by ratio paired *t*-test. Statistical significance was calculated by two-tailed tests, and *p* < 0.05 was considered statistically significant.

## Results

### Incidence of *Candida* species in past 10  years

**Figure 1:**
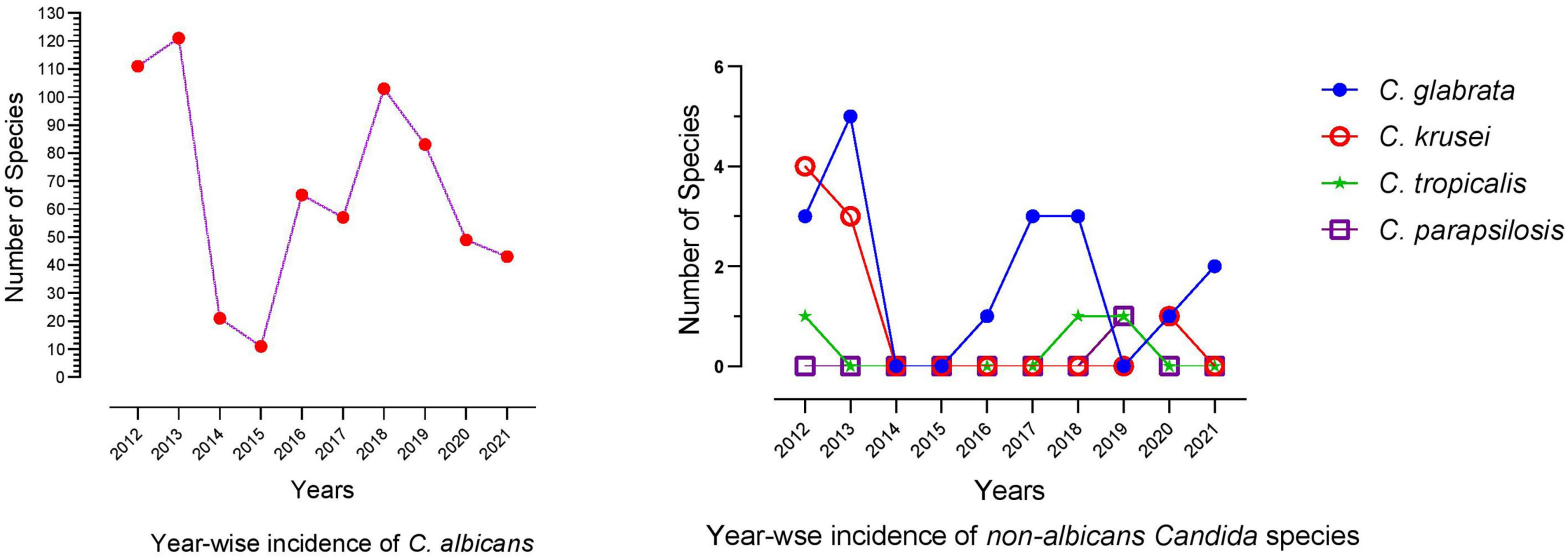
*Candida* species from cutaneous candidiasis reported in the past 10  years (2012–2021).

In the past 10 years, 3,113 patients with cutaneous mycosis were examined by direct microscopy and fungal routine culture, in which 694 (22.29%) were diagnosed with cutaneous candidiasis. Among the candidiasis patients, 545 (78.53%) were male, and 149 (21.47%) were female. Regarding the *Candida* species type, the *C. albicans* were prominently detected in 664/694 (95.68%) patients, while non-*C. albicans* were found only in 30/694 (4.32%) patients. The high number of *C. albicans* were reported in year 2013 (*n* = 121, 18.22%), followed by 2012 (*n* = 111, 16.71%) and 2018 (*n* = 103, 15.51%). Among the 30 non-*C. albicans* species *C. glabrata* were reported in 18/694 (2.59%) patients, *C. krusei* in 8/694 (1.15%), *C. tropicalis* in 3/694 (0.43%), and *C. parapsilosis* was detected only in one patient (*n* = 1/694, 0.14%). The year-wise incidence of *C. albicans* and non-*C. albicans* species are presented in Figure 1.

### Occurrence of *Candida albicans* in different age and gender groups

The median age of patients was 38-year, range (from 8 months to 82 years), interquartile range (30–48 years). Most cases occurred in the adult age group (19–50 years). For *C. albicans* only three (0.45%) cases were reported in infants, and seven (1.05%) were from the pediatric group; three were male, and four were female pediatric patients. From the older adult group, 18 (2.71%) *C. albicans* were isolated, of which 17 were from male patients, and only one was from a female patient. From the adult age group (19–65 years), a total of 636 (95.78%) cases were reported, of which 500/636 (78.61%) were from males, and 136/636 (21.38%) were from female patients. In the current study, we found that the *C. albicans* causing cutaneous candidiasis occurred in a high proportion in males (*n* = 522 out of 664, 78.61%) compared to females (*n* = 142 out of 664, 21.39%; *p*-value = 0.0001). Among the age groups, the adult age group II for males and adult age group I for females were more vulnerable to *C. albicans*. The year-wise occurrence of *C. albicans* in different age groups and gender and their statistical significance (*p*-values) are summarized in Table 1.

**Table 1:** Occurrence of *Candida albicans* in different age groups and gender.

Year	Cases	M:F	Age groups							*p*-value^#^
			< 1	1–18	19–39	31–40	41–50	51–65	> 65	
2012	111	104:07	1	1	39	33	28	8	1	< 0.05
2013	121	115:06	0	0	34	41	25	18	3	< 0.05
2014	21	17:04	0	0	6	9	4	2	0	0.0645
2015	11	9:02	0	0	0	4	4	3	0	0.0815
2016	65	47:18	0	0	14	24	13	11	3	< 0.05
2017	57	46:11	0	0	10	15	18	12	2	< 0.05
2018	103	59:44	0	3	27	29	29	15	0	< 0.05
2019	83	56:27	1	2	21	22	23	10	4	< 0.05
2020	49	37:12	1	0	7	13	17	9	2	< 0.05
2021	43	32:11	0	1	9	11	14	5	3	< 0.05
SUM	664	261:71	3	7	167	201	175	93	18	< 0.05
*p*-value	< 0.05^#^	0.0001^*^								

M:F = Male ratio female.

* Ratio paired *t*-test.

# Student’s *t*-test.

### Occurrence of non-*Candida albicans* in different age and gender groups

The number of non-*C. albicans* (*n* = 30 out of 694, 4.32%) were relatively much smaller than *C. albicans* detected in the present study. Four different types of non-*C. albicans* species were detected, which were *C. glabrata* (*n* = 18 out 30, 60%), *C. krusei* (*n* = 8 out 30, 26.67%), *C. tropicalis* (*n* = 3 out of 30, 10%) and *C. parapsilosis* (*n* = 1 out of 30, 3.33%). The number of non-*C. albicans* isolates from male patients (n = 22 out of 30, 73.33%) were high compared to females (n = 8 out of 30, 26.67%). The number of different non-*C. albicans* species concerning different age and gender groups are presented in Table 2.

**Table 2:** Occurrence of non-*Candida albicans* species in different age groups and gender.

Species	*C. glabrata*	*C. krusei*	*C. tropicalis*	*C. parapsilosis*	SUM
Total cases	18	8	3	1	30
Male:female	2:1	7:1	2:1	1:0	11:4
Age < 1	0	1	0	0	1
Age 19–30	5	3	0	0	8
Age 31–40	1	2	1	0	4
Age 41–50	6	0	0	1	7
Age 51–65	4	2	2	0	8
Age > 65	2	0	0	0	2

### Antifungal susceptibility patterns of *Candida albicans*

For *C. albicans* the lowest antifungal susceptibility was reported for terbinafine, with only 10% of the isolates susceptible out of 157 tested strains. Among the azole class, miconazole showed the lowest susceptibility with 29.32% out of 440 tested isolates, while 40.23% of isolates were intermediate and 30.45% resistant. Besides, itraconazole and fluconazole, two of the most extensively used antifungal drugs, have resistance rates of 16.10 and 9.34%, respectively. Among the other tested antifungal drugs, only two isolates in 2012 were intermediate-resistant to nystatin, while only one isolate in 2021 was found resistant to 5-flucytosine, and none of the isolates exhibited resistance to amphotericin B. The antifungal susceptibility profile of all tested antifungal agents against the *C. albicans* is summarized in Table 3.

**Table 3:** Antifungal susceptibility profile of *C. albicans* for all tested drugs in the past 10  years.

Antifungal agents	Tested isolates	Susceptible (%)	Intermediate (%)	Resistant (%)
5-Flucytosine	170	99.41	0	0.59
Amphotericin B	172	100	0	0
Fluconazole	632	85.28	5.38	9.34
Itraconazole	652	78.53	5.37	16.10
Ketoconazole	490	87.75	9.80	2.45
Miconazole	440	29.32	40.23	30.45
Nystatin	490	99.59	0.41	0
Terbinafine	157	10.83	3.82	85.35
Voriconazole	165	59.39	1.82	38.79

**Figure 2:**
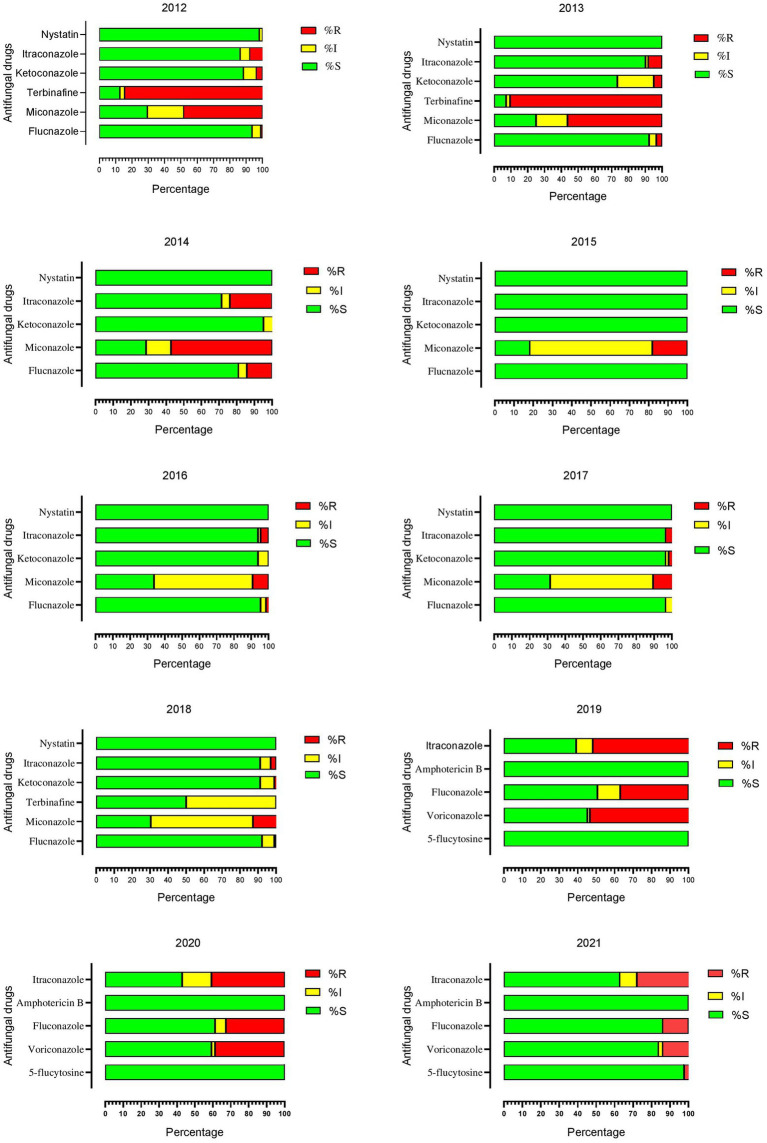
Year-wise antifungal susceptibility patterns of *Candida albicans* against all tested drugs.

The terbinafine was tested in 2012 and 2013 and showed the highest resistance, 84.47, and 90.47%, respectively. In 2014, the highest resistance was reported against miconazole, which was 57.14% out of 21 tested isolates. From 2015 to 2018, the resistance rate was comparatively lower except for the miconazole, which was 18.18% in 2015, 9.23% in 2016, 10.52% in 2017, and 12.74% in 2018. Onward 2019, antifungal susceptibility tests were performed by ATB fungus-2 kit, in which the highest resistance was observed for itraconazole, fluconazole, and voriconazole. In 2019 resistance to itraconazole, voriconazole, and fluconazole were 53.16, 53.42, and 36.98%, in 2020 it was 40.81, 38.77, and 32.65%, while in 2021 it was 27.90, 13.95, and 13.95%, respectively. Year-wise antifungal susceptibility profiles of *C. albicans* against all tested drugs are presented in Figure 2.

### Trend of fluconazole and itraconazole susceptibility in *Candida albicans* over time

**Figure 3:**
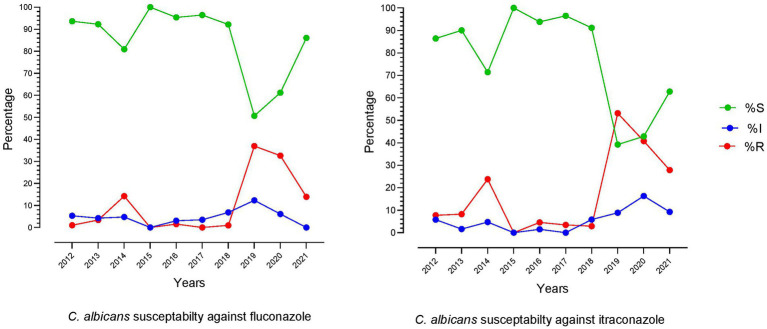
Trend of fluconazole and itraconazole susceptibility patterns of *C. albicans* over the last 10  years (2012–2021).

From a broader perspective, the last 10 years’ fluconazole and itraconazole resistance patterns showed little similarity. However, the combined susceptibility rate of fluconazole was 85.28%, while itraconazole was 78.53%. The lowest resistance rates were noted in 2018 and reached the highest for both drugs in the next 2 years (2019 and 2020). The similarity in pattern between these two drugs indicates that the exact molecular mechanism might be involved in developing resistance against azole drugs. The trend of fluconazole and itraconazole susceptibility patterns over time is shown in Figure 3.

### Antifungal susceptibility pattern of non-*Candida albicans* species

The *C. parapsilosis* showed resistance to fluconazole, itraconazole, and voriconazole and was susceptible to amphotericin B and 5-flucytosine. Among the three *C. tropicalis* isolates, resistance to terbinafine and miconazole were observed in only one isolate. For *C. krusei,* 80% of isolates were resistant to terbinafine out of five tested strains, while all other isolates were susceptible to amphotericin B and 5-flucytosine. For *C. glabrata,* one isolate detected in 2021 was resistant to 5-flucytosine; however, it was susceptible to amphotericin B and all tested azole drugs. Similarly, another isolate of *C. glabrata* reported in 2013 showed resistance to nystatin, the only nystatin-resistant isolate in the current study. The antifungal susceptibility profile of *C. glabrata* is summarized in Table 4.

**Table 4:** Antifungal susceptibility profile of *C. glabrata* for all tested drugs in the past 10 years.

Antifungal agents	Tested isolates	Susceptible (%)	Intermediate resistant (%)	Resistant (%)
5-Flucytosine	3	66.67	0	33.33
Amphotericin B	3	100	0	0
Fluconazole	17	88.24	11.76	0
Itraconazole	18	100	0	0
Ketoconazole	12	91.67	8.33	0
Miconazole	12	50	41.67	8.33
Nystatin	15	93.33	0	6.67
Terbinafine	5	20	0	80
Voriconazole	3	100	0	0

## Discussion

The prevalence of cutaneous candidiasis varies regarding the geography, demography of patients, type of the fungal pathogen, and many other environmental factors (Kühbacher et al., 2017). In the current study, the prevalence rate of cutaneous candidiasis over the past decade was 22.29% which makes it different from other regions of the world, where it is reported to be 40.5% in Iran, 57% in Serbia, and 82.9% in Brazil (de Albuquerque Maranhão et al., 2019; Otašević et al., 2019; Khodadadi et al., 2021). The difference in the prevalence might be due to different environmental conditions and the social status of the populations (Zareshahrabadi et al., 2021). In the present study, the infection rate was highly reported in the male population (78.53%) compared to females (21.47%). In different countries, gender-based prevalence varies; a previous study from China, Italy, and France reported an equal proportion of males and females infected with cutaneous mycosis (Vena et al., 2012; Cai et al., 2016; Faure-Cognet et al., 2016). Studies from South Korea and Chile reported a high proportion of *Candida*-infected females (Cruz Ch et al., 2011; Yoon et al., 2014). However, a study from Iran showed resemblance to our finding, with high cases of cutaneous candidiasis among males (Zamani et al., 2016). These contradictions depend on differences in occupational activities, personal hygiene, and exposure to contamination of male and female populations (de Albuquerque Maranhão et al., 2019). In our study, cutaneous candidiasis mainly occurred in the adult age group (from 19 to 50 years). Some other studies reported that patients below 20 are more vulnerable to candidiasis; however, the studies from Iran, South Korea, and India agreed with our findings (Nawal et al., 2012; Yoon et al., 2014; Cai et al., 2016; Khodadadi et al., 2021). The main reason for the adult age group’s link with cutaneous candidiasis might be that these populations have more involvement in job markets and social activities with a high chance of exposure to *Candida* infections (Khodadadi et al., 2021).

More than 200 *Candida* species have been identified, of which over 15 are known for human pathogenicity, among which the *C. albicans* are highly reported (Palese et al., 2018). Similarly, in our study, 95.68% of cutaneous candidiasis was caused by *C. albicans*. The high infection rate of *C. albicans* is due to its ability to grow in different morphological forms like true hyphae, pseudo-hyphae, and unicellular budding yeast, which enhance its virulence and invading host cell activity (Nam et al., 2022). Moreover, underlying diseases, immunosuppressive states, antibiotic therapy, and skin environment variation are the factors due to which the commensal *C. albicans* switched into a true pathogen (Palese et al., 2018). In the present study, the *C. glabrata* was detected in 18 cases, the highest among non-*C. albicans* species. This differs from the studies reported in Cameroon, Nigeria, and India, where *C. tropicalis* are more prevalent than *C. glabrata* (Verma et al., 2021). However, a similar drift was observed in North America and many European countries (Song et al., 2020). After *C. glabrata*, the *C. krusei* was reported as the second high in number among the non-*C. albicans* species. It is a matter of concern because *C. krusei* is one of the multidrug-resistant species and is intrinsically resistant to fluconazole (Jamiu et al., 2021).

In the current study, the *C. albicans* show high resistance to terbinafine, i.e. 85.35% of 157 tested isolates. Similarly, for *C. glabrata* and *C. krusei*, 80% of the isolates were resistant to terbinafine. This high resistance might be due to the weak inhibitory activity of terbinafine against all *Candida* species except *C. parapsilosis* (Ameen et al., 2014; Noguchi et al., 2019). In the azole class, the high resistance (30.45%) was reported for miconazole, while 40.23% of 440 tested *C. albicans* isolates were intermediated resistant. The high resistance to miconazole is due to its improper usage as a topical therapeutic agent for cutaneous candidiasis (Taudorf et al., 2019).

Similarly, high resistance was reported for voriconazole; 38.79% out of 175 tested isolates. For fluconazole and itraconazole, the resistant rate was 9.34 and 16.10%, while 5.38 and 5.37% of the isolate were intermediate resistant, respectively. In this study, the azoles are comparatively less susceptible than polyenes and flucytosine. The high resistance to azole might be due to its inappropriate usage in agriculture and clinical settings in China (Zhou et al., 2022). Moreover, the fungistatic nature of azole drugs imposes a robust direct selection of antifungal-resistant species (Das et al., 2019). Only one isolate of *C. albicans* and *C. glabrata* show resistance to 5-flucytosine. For nystatin, only one *C. glabrata* isolate was resistant, and two *C. albicans* were intermediate resistant, while none of the isolates was resistant to amphotericin B. According to a report, a single antifungal agent and corticosteroid drugs are good options for curing cutaneous candidiasis (Taudorf et al., 2019). Hence, based on our findings, we suggest nystatin, a topical antifungal agent, and a corticosteroid drug for curing cutaneous candidiasis in our regions.

Moreover, it is suggested that the general population not to take antifungal drugs without a proper diagnosis of mycotic infections and prescriptions from a dermatologist. The laboratory screening of candidiasis needs to be performed molecularly or by MALDI TOF MS to correctly identify *Candida* species (Zhao et al., 2018). Furthermore, national guidelines for treating cutaneous mycotic infections need to be developed for proper medication and to halt the incidence of antifungal-resistant pathogens.

The current study has some limitations; our study was based on a single center in the eastern Guangdong province. Hence our findings might not be generalized to other regions because the prevalence of cutaneous candidiasis varies due to environmental and socio-economic factors (Dhillon and Chopra, 2022). Moreover, our study was based on the available laboratory records; therefore, clinical and detailed demographic features were not analyzed. To provide new insights, from now on, we intend to collect the clinical and patient demographic data for future research work and scientific base treatment of cutaneous candidiasis. On a vaster glimpse, the premise of this study entails significant epidemiological findings that are valuable for scheming approaches to improve the management of cutaneous candidiasis.

## Conclusion

In the current study, we summarized the significant updated data about the prevalence of cutaneous candidiasis, species distribution, and antifungal susceptibility patterns of *Candida* species. Over the past decade of surveillance, *C. albicans* was a primary cause of cutaneous candidiasis, and resistances to terbinafine and azole were prominent. The *C. glabrata* were reported in high number among the few non-*C. albicans* isolates. Amphotericin B and 5-flucytosine were more susceptible drugs in the last 3 years. Over the years, nystatin has shown excellent activity against all *Candida* species. National trends of antifungal susceptibility and continuous monitoring are needed. The epidemiological outcomes of the present study will provide baselines for more in-depth research and help healthcare officials to tackle the challenges of antifungal resistance.

## Data availability statement

The raw data supporting the conclusions of this article will be made available by the authors, without undue reservation.

## Ethics statement

The studies involving human participants were reviewed and approved by the Ethics Committee of Skin and Venereal Diseases Prevention and Control Hospital of Shantou city, Guangdong, China. The ethics committee waived the requirement of written informed consent for participation.

## Author contributions

HB and YZ: study idea and plan. HB and BH: attainment of data. HB, MS, and XC: analysis and interpretation of data. HB, MAS, and YZ: drafting of the manuscript. YZ and MAS: critical revision of the manuscript for important intellectual content. YZ: administrative, technical, material support, and institutional study supervision. All authors contributed to the article and approved the submitted version.

## Funding

This study is funded by the Second Affiliated Hospital of Shantou University Medical College to HBil and his mentor YZ.

## Conflict of interest

The authors declare that the research was conducted in the absence of any commercial or financial relationships that could be construed as a potential conflict of interest.

## Publisher’s note

All claims expressed in this article are solely those of the authors and do not necessarily represent those of their affiliated organizations, or those of the publisher, the editors and the reviewers. Any product that may be evaluated in this article, or claim that may be made by its manufacturer, is not guaranteed or endorsed by the publisher.
